# Plasma rotavirus-specific IgA and risk of rotavirus vaccine failure in infants in Malawi

**DOI:** 10.1093/cid/ciab895

**Published:** 2021-11-12

**Authors:** Louisa Pollock, Aisleen Bennett, Khuzwayo C. Jere, Jonathan Mandolo, Queen Dube, Naor Bar-Zeev, Robert S. Heyderman, Nigel A. Cunliffe, Miren Iturriza-Gomara

**Affiliations:** 1 Centre for Global Vaccine Research, Institute of Infection, Veterinary and Ecological Sciences, University of Liverpool, Liverpool, UK; 2 Malawi Liverpool Wellcome Trust Clinical Research Programme, Kamuzu University of Health Sciences, Blantyre, Malawi; 3 Department of Biomedical Sciences, Kamuzu University of Health Sciences, Blantyre, Malawi; 4 Department of Paediatrics, Kamuzu University of Health Sciences, Blantyre, Malawi; 5 International Vaccine Access Center, Dept. International Health, Bloomberg School of Public Health, Johns Hopkins University, Baltimore, USA; 6 Research Department of Infection, Division of Infection and Immunity, University College London, London, UK; 7 National Institute for Health Research Health Protection Research Unit in Gastrointestinal Infections at University of Liverpool

**Keywords:** rotavirus, IgA, vaccine, Malawi, gastroenteritis

## Abstract

**Background:**

Rotavirus vaccine efficacy is reduced in low-income populations, but efforts to improve vaccine performance are limited by lack of clear correlates of protection. While plasma rotavirus (RV)-specific IgA appears strongly associated with protection against rotavirus gastroenteritis in high-income countries, weaker association has been observed in low-income countries. We tested the hypothesis that lower RV-specific IgA is associated with rotavirus vaccine failure in Malawian infants.

**Methods:**

In a case-control study we recruited infants presenting with severe rotavirus gastroenteritis following monovalent oral rotavirus vaccination (RV1 vaccine failures). Conditional logistic regression was used to determine the odds of rotavirus seronegativity (RV-specific IgA<20 U/mL) in these cases compared 1:1 with age-matched, vaccinated, asymptomatic community controls. Plasma RV-specific IgA was determined by ELISA for all participants at recruitment, and for cases at 10 days post symptom onset. Rotavirus infection and genotype were determined by antigen testing and RT-PCR respectively.

**Results:**

In 116 age-matched pairs, infants with RV1 vaccine failure were more likely to be RV-specific IgA seronegative than controls: OR 3.1 (95%CI 1.6-5.9), p=0.001. In 60 infants with convalescent serology, 42/45 (93%, 95%CI 81-98%) infants seronegative at baseline became seropositive. Median rise in RV-specific IgA concentration following acute infection was 112.8 (IQR 19.1-380.6) fold.

**Conclusions:**

In this vaccinated population with high residual burden of rotavirus disease, RV1 vaccine failure was associated with lower RV-specific IgA, providing further evidence of RV-specific IgA as a marker of protection. Robust convalescent RV-specific IgA response in vaccine failures suggests differences in wild-type and vaccine-induced immunity, which informs future vaccine development.

## Background

Introduction of rotavirus vaccines into childhood immunization programmes has reduced global child deaths from diarrhoeal disease[1], but current vaccines are less effective in low-income, high-mortality countries than in higher income settings[2]. Multiple explanations for this disparity have been proposed, including factors which may inhibit the initial vaccine response[3–6] and factors which may increase rotavirus exposure or increase susceptibility to rotavirus disease in later infancy[7–9]. Efforts to assess and improve rotavirus vaccines have been hampered by the lack of a proven correlate or surrogate marker of protection against rotavirus disease[10]. Biomarkers associated with protection are required to reduce the need for very large scale clinical trials focussed on increasingly rare clinical outcomes[10].

In studies of immune response to natural rotavirus infection, plasma rotavirus (RV)-specific IgA levels were shown to increase with repeated exposure to natural infection, and were associated with reduced risk of future rotavirus disease[11]. Higher concentrations of RV-specific IgA, and in some studies IgG, were associated with reduced likelihood of severe rotavirus disease in early case-control studies[12]. In studies of response to oral rotavirus vaccines, post-immunisation RV-specific IgA has been correlated with vaccine efficacy at population level[13, 14]. At individual level, in a recent pooled analysis of monovalent oral rotavirus vaccine (RV1) trial data, Baker et al.[15] demonstrated that RV-specific IgA seroconversion (defined as post-immunisation RV-specific >20 U/mL) was strongly associated with protection against rotavirus gastroenteritis. However, while seroconversion and RV-specific IgA titers post-immunisation were very strongly associated with protection in low child mortality countries, a much weaker association was observed in high-child mortality countries. Similarly, Lee et al.[16] found that post-immunisation RV-specific IgA was a sub-optimal correlate of protection against rotavirus gastroenteritis in infants in Bangladesh. The extent to which plasma RV-specific IgA may be a mechanistic correlate of protection, causally responsible for observed protection, or a non-mechanistic proxy marker of an alternative, likely mucosal, protective immune response is debated[17, 18]. Individual data on RV-specific IgA measured at time of presentation with rotavirus gastroenteritis would add new information to further inform this debate.

Malawi is a low-income country with high rotavirus burden and relatively low (~ 50%) rotavirus vaccine efficacy[19], which introduced RV1 nationally in 2012, with vaccine coverage reaching 95% by 2015[20]. We previously demonstrated that low serum RV-specific IgA is associated with increased rotavirus load in vaccinated Malawian children with acute gastroenteritis[21]. We now test the hypothesis that low RV-specific IgA is associated with increased risk of clinical rotavirus vaccine failure.

## Methods

This was a prospective case: control study. Ethical approval was granted by the University of Malawi College of Medicine (P.09/14/1624) and University of Liverpool (00758) Research Ethics Committees. All participants were recruited following informed consent from a parent/guardian.

Infants presenting with symptoms of gastroenteritis to three primary healthcare centres and a referral hospital in Blantyre, Malawi between January 2015 and January 2017 were screened for recruitment within the local diarrhoeal surveillance platform[20]. Cases (vaccine failures) met the following eligibility criteria: aged between 10 weeks and 1 year; received 2 doses of oral RV1 documented in their hand-held health record; severe gastroenteritis, defined as Vesikari score ≥11[22]; and rotavirus positive by rapid stool immunochromatography test (RotaStrip®, Coris Bioconcept, Belgium).

Age-matched community controls were recruited in a 1:1 ratio. GPS locations were randomly generated (using R 3.1.1, The R Foundation for Statistical Computing) within recruitment site healthcare catchment areas to search for controls. Controls met the following eligibility criteria: born within ±30 days of matched case; received two doses of RV1; and reported no diarrhoea within one week prior to recruitment.

### Data collection and anthropometry

Demographic and socio-economic data were collected by structured interview. Nutritional status was determined by measurement of weight, length and mid-upper arm circumference (MUAC), compared to WHO age-determined z scores[23]. Infants with weight for height z score <-3SD and infants over six months old with a MUAC <11.5cm were considered to have severe acute malnutrition[24]. Weight of cases with signs of some or severe dehydration noted on admission was adjusted by 5 or 10% respectively[25]. Z-score restrictions were applied in accordance with WHO guidelines so that biologically implausible outliers were removed[26].

### Sample collection

All participants had stool, saliva and plasma samples collected at time of presentation with gastroenteritis (cases) or recruitment (controls). Rotavirus gastroenteritis cases had a convalescent plasma sample taken 10 days following onset of diarrhoea, corresponding to the peak rise in RV-specific IgA following acute infection in infants[27].

### Laboratory methods

For detailed laboratory methods see Supplementary Methods. Nucleic acid was extracted from stool from gastroenteritis cases and community controls using the Qiagen Viral RNA Mini-Kit (Qiagen, Germany). Reverse transcription using random primers was used to generate complementary DNA[28]. VP6 qRT-PCR was used detect rotavirus. Community controls with VP6 ≥100 copies/ml by qRT-PCR were considered to have asymptomatic rotavirus infection, those with VP6<100copies/ml (analytical sensitivity) were considered rotavirus negative. Genotyping was undertaken by hemi-nested PCR and gel electrophoresis was undertaken for all rotavirus positive samples possessing a qRT-PCR cycle threshold below 35[29].

Plasma RV-specific IgA was determined by antibody-sandwich ELISA[30]. Quantification was made by comparison to a standard plasma[31], reported as geometric mean concentration (GMC) in units per millilitre (U/mL). RV-specific IgA seropositivity was defined as a GMC >20U/mL[32].

HBGA phenotyping was determined as described in Pollock et.al, 2019[5]. In brief, antigens A, B, H, and Lewis a and b were detected in saliva by ELISA, using specific monoclonal antibodies, detected by peroxidase conjugated anti-mouse IgM. Infants with detectable salivary A, B or H antigen were classified as secretors. Where detection of A, B and H antigens was negative or borderline, secretor status was confirmed by ELISA to detect lectin antigen[33].

### Statistical Analysis

All statistical analysis was performed in StataIC version 13.1 (StataCorp, US). Summary statistics for demographics, nutritional status and socio-economic factors were reported for cases and controls. Continuous variables were compared by t-test for parametric and Wilcoxon rank-sum test for non-parametric data. Categorical variables were compared by Chi-squared test, or Fisher’s exact test where cell values were less than 10.

The primary outcome measure was the odds of RV-specific IgA <20U/mL in cases compared to controls calculated by conditional logistic regression. With 1:1 controls, a sample size of 137 cases was estimated to achieve 80% power to detect an odds ratio of 2.0 (alpha 0.05), assuming 50% RV-specific IgA seropositivity in controls (assumption based on vaccine trial data[19]). In addition, comparison of RV-specific IgA as a continuous variable between cases and controls was made by Wilcoxon rank sum test. Reciever operating characteristics (ROC) analysis was used to evaluate the utility of RV-specific IgA concentration in discriminating between vaccine failures and community controls. A cutoff point of RV-specific IgA concentration that best predicted case-control status was determined by maximising Youden’s index ((sensitivity+specificity)-1)[34, 35].

## Results

A total of 196 rotavirus positive infants meeting eligibility criteria were identified. 76 infants declined to participate, mainly due to the requirement for blood sampling. A total of 120 severe rotavirus gastroenteritis cases were therefore recruited.

The median age of presentation of cases was 9.0 months (IQR 7.6-10.6 months). The median age at recruitment for community controls was 9.8 months (IQR 8.3-11.1 months). The median Vesikari score was 14 (IQR 13-16).

Genotype was determined for 116/120 (97%) rotavirus gastroenteritis cases. Four genotypes accounted for over 75% of total genotypes: G1P[8] (32%), G2P[4] (26%), G12P[6] (10%) and G2P[6](9%).

There were no significant differences between cases and controls in weight or height for age Z scores, sanitation or socioeconomic factors, HIV-exposure, male gender or low birth weight (Table 1). As previously reported[5], cases were significantly less likely than controls to have non-secretor HBGA phenotype.

### Comparison of RV-specific IgA between RV1 vaccine failures and community controls

RV-specific IgA was determined for 117 rotavirus gastroenteritis cases (vaccine failures) and 119 community controls. RV-specific IgA was generally low in this population: 55% of community controls were seronegative (RV-specific IgA <20U/mL) (Table 2). There was no difference in baseline RV-specific IgA by non-secretor HBGA phenotype (Supplementary Table 1). In those with detectable RV-specific IgA, RV-specific IgA concentration was significantly lower in RV1 vaccine failures compared to controls (Table 2). In 116 age-matched pairs, the odds of being seronegative were over three times higher in vaccine failures compared to controls: OR 3.1 (95%CI 1.6-5.9), p=0.001.

In ROC analysis, the inverse of the RV-specific IgA concentration showed some utility in discriminating between vaccine failures and controls, with an area under the curve of 0.61 (95%CI 0.54-0.68) (Supplementary Figure 1).

### RV-specific IgA response to natural infection in RV1 vaccine failures

Paired presentation and convalescent plasma samples were available for 60/120 (50%) rotavirus gastroenteritis cases. The median number of days after illness onset at convalescent sampling was 10 (IQR 9-12). The distribution of baseline RV-specific IgA was similar in those with convalescent serology data available to those without (Supplementary Table 2). Of 45 infants seronegative at presentation, 42 (93%, 95%CI 81-98%) were seropositive at follow-up. The follow-up GMC was significantly higher than baseline (Wilcoxon matched-pairs signed rank test, p<0.0001) (Table 3). The median convalescent rise in RV-specific IgA concentration was 112.8 (IQR 19.1-380.6) fold.

## Discussion

In this high mortality, high disease burden population in Malawi, lower RV-specific IgA measured at time of disease presentation was strongly associated with increased odds of RV1 clinical vaccine failure. This provides further evidence that RV-specific IgA is associated with clinical protection at individual, as well as population, level. Our findings are consistent with a post-hoc analysis of data from a Phase III efficacy trial of RV1 conducted in Malawi and South Africa, where post-immunisation RV-specific IgA seropositivity was associated with reduced risk of subsequent severe rotavirus gastroenteritis (OR 0.39 (95%CI 0.29-0.52), p<0.001)[14]. Our data is also consistent with a large pooled analysis of RV1 trial data across 16 countries, which showed that higher post-immunisation RV-specific IgA levels were associated with reduced cumulative incidence of severe rotavirus gastroenteritis[15].

Using ROC analysis, RV-specific IgA concentration was shown to have relatively limited utility in discriminating between RV1 vaccine failures and controls in this population. This could reflect the limitations of the case-control design, where some community controls could also be at risk of vaccine failure if exposed to infection, and where some cases could already have had rising IgA in response to acute infection. However, it could also support the hypothesis that RV-specific IgA is a surrogate marker, rather than an absolute correlate, of protection[10, 18]. The extent to which RV-specific IgA is associated with protection across populations varies: Baker et al.[15] found that for any given threshold for IgA, greater protection was conferred in low child mortality countries compared to high child mortality countries, but were unable to identify specific confounding factors which explained this difference. Nevertheless, they concluded that a seroconversion threshold of 20U/mL remained a practical and informative measure of protection.

Lee et al.[16] found that uniformly poor RV-specific IgA responses post-immunisation in infants in Bangladesh, together with rapidly waning immunity in some infants, reduced the utility of RV-specific IgA as a correlate of protection. Post-immunisation RV-specific IgA responses in Malawian infants are similarly poor compared to higher income countries. In a recent Malawian birth cohort, we found only 24% of infants seroconverted[5]. Despite this, we demonstrate that most infants with clinical rotavirus vaccine failure show a robust RV-specific IgA response to natural infection. Almost all infants demonstrated a significant rise in RV-specific IgA titres within 10 days of symptom onset, similar to the immune response seen in unvaccinated infants[27, 36]. In this population, 93% of infants seronegative at baseline seroconverted following acute infection. Of note infants with low RV-specific IgA at baseline, and of similar age to our study population, show a less consistent response to challenge with “booster” live, oral rotavirus vaccination. Thus in Mali, a booster (4^th^) dose of oral pentavalent rotavirus vaccine (RV5) at age nine months resulted in seroconversion in 56.9% of infants who were seronegative at baseline[37], whereas in Bangladesh only 43.6% of baseline seronegative infants seroconverted following receipt of a 3^rd^booster dose of RV1 [38]. Lower immune responses to booster doses of vaccine compared with natural infection could reflect continuing vaccine-specific inhibitory factors persistent in later infancy, or may reflect a higher virus inoculum in natural infection. There is evidence from early studies of RV1 and of the live rotavirus vaccine ORV-116E that higher vaccine doses generate a more robust immune response[39–41].

A key strength of our study is that we measured RV-specific IgA at time of presentation with severe gastroenteritis. The main limitation is that the cross-sectional case-control design means that we are unable to determine whether low RV-specific IgA at time of presentation relates to poor initial response to vaccination, or to waning immunity. Rotavirus vaccine effectiveness appears to be lower in the second year of life in some low-income countries, but the extent to which this may reflect waning immunity is unclear[42]. This is an important distinction, as it could determine whether efforts should continue to focus on improving magnitude and duration of initial vaccine response, or designing optimal vaccine booster strategies. A cohort study determining the dynamics of RV-specific IgA response from vaccination throughout the first year of life could address this issue.

This study provides data in a rotavirus vaccinated population of RV-specific IgA measured at time of rotavirus clinical vaccine failure. Further data from diverse populations are required to determine whether a particular level of RV-specific IgA can be considered broadly protective, or at least highly predictive of protection against clinical vaccine failure. These data take us closer to the indentification of a reliable protective threshold would greatly assist efforts to improve the performance of current vaccines, and the evaluation of new vaccines.

## Supplementary Material

Supplementary data

## Figures and Tables

**Table 1:** Host and socio-economic factors: RV1 vaccine failures and community controls

Characteristic^a^	RV1 vaccine failures	Community Controls	p
**Infant Characteristics**			
Male	73, 61% (52-69%)	61, 51% (42-60%)	0.12^b^
HIV-exposed^c^	17, 14%(9-22%)	19, 16%(10-24%)	0.72^b^
Low birth weight (<2.5kg)	14/112 13% (8-20%)	12/118 9%(5-16%)	0.44^b^
Non secretor	14/119 12% (7-19%)	34/120 28% (21-37%)	0.001^b^
**Nutritional Status**			
Median weight for age z-score (IQR)^d^	-0.37 (-1.39-0.45)	-0.42(-1.0-0.36)	0.88^e^
Median length for age z-score (IQR)	-0.68(-1.75-0.95)	-0.76 (-1.98- -0.06)	0.19^e^
Median weight for length z-score (IQR)^d^	-0.62(-1.58-0.37)	-0.05(-1.32-0.88)	0.11^e^
**Sanitation and socioeconomic factors**			
Median household size (IQR)	5 (4-6)	4 (3-6)	0.13^e^
Non-piped water source	22, 19% (12-27%)	17, 14% (9-22%)	0.37^b^
Time to access water			0.52^b^
<5minutes	27, 23% (16-31%)	20, 17% (11-25%)	
5-30 minutes	52, 44% (35-53%)	52, 44% (36-54%)	
>30 minutes	40, 34% (26-43%)	45, 38% (30-48%)	
Pit-latrine type toilet	116, 97% (91-99%)	115, 96% (90-98%)	0.73^b^
Electricity at home	61, 50% (42-59%)	55, 46% (37-55%)	0.44^b^
One or more household members with salary	82, 68% (59-76%)	76, 63% (54-72%)	0.41^b^
Household food insecurity	40, 33% (25-42%)	36, 30% (22-39%)	0.58^b^
Median age of head of household in years (IQR)	30 (26-37)	30 (27-34)	0.40^e^
Median years of maternal education (IQR)	8 (5-11)	9 (7-11)	0.32^e^

a All proportions reported as number (proportion, 95% confidence interval of proportion). Denominator for all proportions n=120 for both cases and controls unless stated otherwise.

b Chi squared test

c 11/17 HIV exposed RV GE cases and 15/19 HIV exposed community controls had a negative HIV DNA PCR at 6 weeks old. One community control was known HIV infected and on ART. Status of remaining HIV exposed infants was unknown.

d Weight adjusted for dehydration status by adding 5% for some dehydration and 10% for severe dehydration.

e Wilcoxon rank-sum test.

**Table 2:** Comparison of RV-specific IgA among RV1 vaccine failures and community controls

	RV1 vaccine failures	Community controls	p
**Undetectable RV-specific IgA**	55/117	41/119	0.05^a^
n, % (95%CI)	47 (38-56) %	34 (26-44) %	
**RV-specific IgA <20 U/mL**	89/117	66/119	0.001^a^
n, % (95%CI)	76 (67-83) %	55 (46-64) %	
**RV-specific IgA concentration^b^**	18.1 (7.1-53.1) U/mL	48 (14.5-146.7) U/mL	0.01^c^
Median (IQR) U/mL			

a Chi squared test

b In infants with detectable RV-specific IgA

c Wilcoxon rank sum test

**Table 3:** RV-specific IgA response to natural infection in RV1 vaccine failures

	RV1 vaccine failures at presentation^a^	RV1 vaccine failures convalescent sample
**Undetectable RV-specific IgA**	29/60	1/60
n, % (95%CI)	48 (36-61) %	2 (0.2-11) %
**RV-specific IgA <20U/ml**	45/60	3/60
n, % (95%CI)	75 (62-85) %	5 (2-15) %
**RV-specific IgA concentration^b^**	18.8 (6.4-54.7) U/mL	433.5 (130.3-896.4) U/mL
Median (IQR) U/mL		

a RVGE cases with convalescent sample available.

b infants with detectable RV-specific IgA

## Summary

In a case-control study of monovalent rotavirus vaccinated Malawian infants, low plasma rotavirus (RV)-specific IgA at presentation with gastroenteritis was strongly associated with clinical vaccine failure. Infants with vaccine failure nevertheless demonstrated a robust RV-specific IgA response to acute infection.
