# Supplementary material for: Plasma rotavirus-specific IgA and risk of rotavirus vaccine failure in infants in Malawi
Source: Clin Infect Dis. Author manuscript; Available in PMC 2022 Aug 27. (PMC9402641; doi:10.1093/cid/ciab895)

Supplementary Information

Supplementary Methods

VP6 PCR

Primers and probes used in VP6 qRT-PCR and NSP2 are detailed in Supplementary Methods Table 1. Reaction mix was prepared with 12.5µl PCR Mastermix (Low Rox), 0.5µl of each primer (at 20pmol/ µl), 0.25µl probe (20µM), 8.75µl nuclease free water. Reaction mix (22.5 µl) and cDNA(2.5µl) were added to a 96 well FAST plate. Cycling conditions were: 95°C for 2 minutes, then 40 cycles of 95°C for 15 seconds, 60°C for 1 minute.

Supplementary Methods Table 1: Probes and primers for VP6 qRT-PCR and NSP2 RT-PCR

Primer/Probe	Sequence (5'-3')	Nucleotide Positions
**VP6F**	GAC GGV GCR ACT ACA TGG T	747-766
**VP6R**	GTC CAA TTC ATN CCT GGT G	1126-1106
**VP6Probe**	^{FAM} CCA CCR AAY ATG ACR CCA GCN GTA^{MGB}	912-935

Rotavirus serology

RV-specific IgA was determined by sandwich ELISA [26]. Rotavirus (WC3-infected MA104 cell culture lysates) was bound with rabbit anti-rotavirus IgG (provided by Christian Medical College, Vellore, India). Uninfected MA104 cell lysates were included for background correction. Rotavirus-specific IgA was detected using biotin-conjugated rabbit anti-human IgA, with avidin-biotin-peroxidase complex and peroxidase substrate. Quantification was made by comparison to a standard plasma [27] and reported as geometric mean concentration (GMC) in units per litre (U/mL).

Supplementary Table 1: Comparison of baseline RV-specific IgA in infants with and without convalescent serology

	Convalescent serology available	No convalescent serology	p
**Detectable RV-specific IgA**	31/60, 52% (39-64%)	31/57, 54% (41-67%)	0.77
**RV-specific IgA >20U/mL**	15/60, 25% (15-38%)	13/57, 23% (13-36%)	0.78

Supplementary Table 2: Baseline RV-specific IgA by HBGA phenotype

	**Non-secretor** n=48[a]	**Secretor** n=187[b]	**p**
**Undetectable RV-specific IgA** n, % (95%CI)	16, 33% (21-48%)	80, 43% (36-50%)	0.24[c]
**RV-specific IgA <20 U/mL** n, % (95%CI)	30, 63% (48-75%)	125, 67% (60-73%)	0.57[c]
**RV-specific IgA concentration[d]** Median (IQR) U/mL	22.9 (6.9-70.8) U/mL	31.9 (10.9-112-5) U/mL	0.40[e]

a.14/116 cases and 34/119 controls with baseline IgA data were non-secretors. b. 102/116 cases and 85/119 controls with baseline IgA data were secretors. c. Chi squared test. d. In infants with detectable RV-specific IgA. e.Wilcoxon rank sum test.

ROC analysis

In ROC analysis, the inverse of the RV-specific IgA concentration showed some utility in discriminating between vaccine failures and controls, with an area under the curve of 0.61 (95%CI 0.54-0.68) (Figure 1). The point that maximised the Youden index was at an RV-specific IgA concentration of 19.9 U/mL. Sensitivity of discrimination between vaccine failures and controls at this cut-point was 76%, with a specificity of 45%.

Figure 1: ROC curve

Reciever operating characteristics (ROC) curve created with case-control status as the reference variable, and 1/rotavirus-specific IgA concentration as the classification variable.

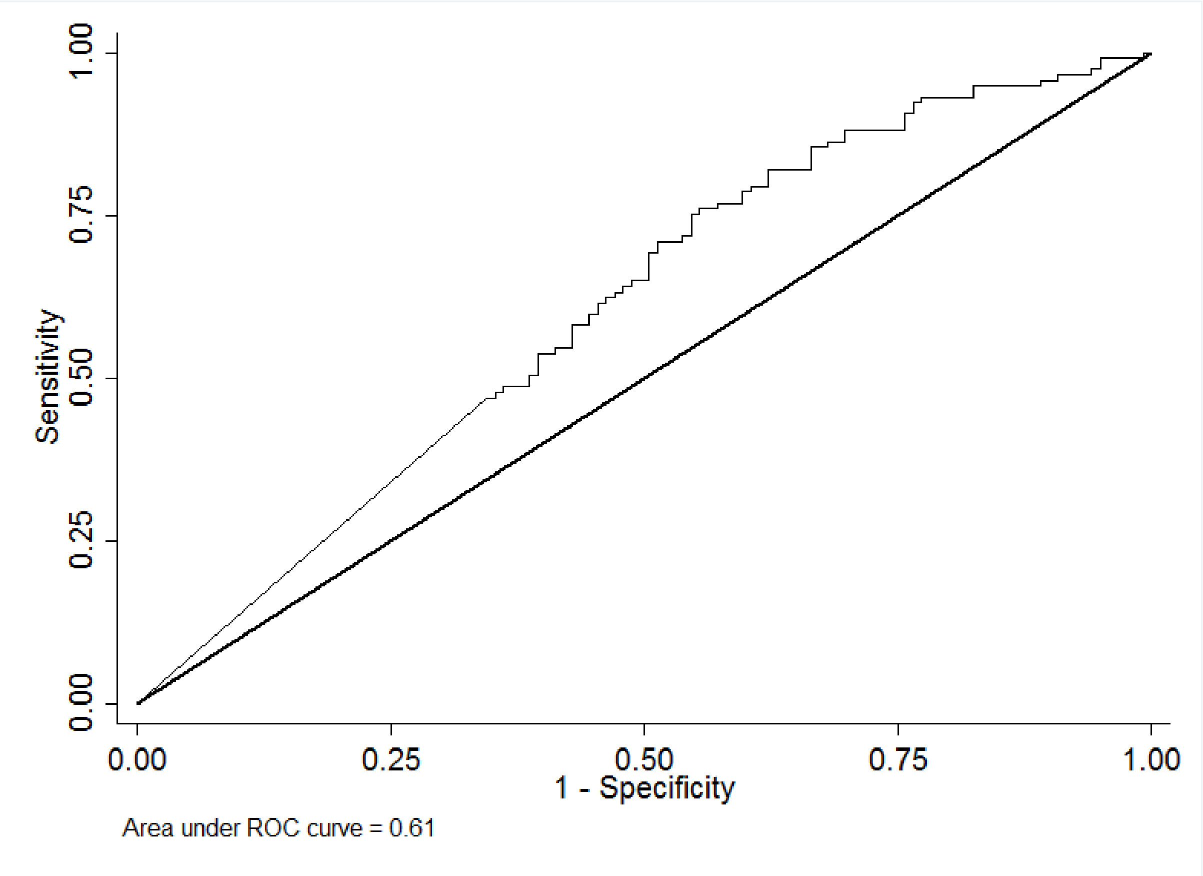

Supplement: Supplementary data [file EMS139532-supplement-Supplementary_data.pdf]
